# Impact of high frequency electromagnetic radiation on bacterial survival and antibiotic activity in exposed bacteria

**DOI:** 10.1038/s41598-025-90599-8

**Published:** 2025-03-06

**Authors:** Ehab A. Hegazy, May A. El-Antrawy

**Affiliations:** 1 Basic Science Department, Delta University for Science and Technology, Gamasa, 11152 Egypt; 2 Microbiology and Immunology Department, Faculty of Pharmacy, Delta University for Science and Technology, Gamasa, 11152 Egypt; 3 Department of Microbiology and Immunology, Faculty of Pharmacy, Delta University for Science and Technology, International Coastal Road, P.O. Box +11152, Gamasa, Mansoura, Dakahlia Egypt

**Keywords:** Extremely high-frequency electromagnetic field, Antibiotics, Bacterial sensitivity, Optical density, Growth rate, Biophysics, Microbiology, Physics

## Abstract

High-frequency electromagnetic waves (HFEMWs) have been shown to influence cellular functions, including bacterial behavior, potentially affecting growth and antibiotic sensitivity. This study evaluated the response of *Escherichia coli* and *Staphylococcus aureus* to HFEMWs across a frequency range of 900 MHz to 73 GHz. The Bacterial sensitivity to antibiotics, including ceftazidime, ceftaroline, gentamycin, doxycycline, and ciprofloxacin, was assessed. The growth rate was evaluated by measuring the optical density (OD) and the number of colony-forming units (CFUs). Our results revealed significant electromagnetic interference (EMI) effects at frequencies of 51.8 GHz and 53 GHz, with 53 GHz showing the most pronounced impact. These frequencies enhanced bacterial susceptibility, with previously resistant *E. coli* and *S. aureus* strains becoming sensitive to tested antibiotics. Conversely, 70.6 GHz and 73 GHz frequencies showed limited effects, while exposure to 900 MHz and 1800 MHz caused no notable changes. These findings highlight the frequency-dependent effects of HFEMWs on bacterial viability and antibiotic sensitivity. This research underscores the potential of HFEMWs as a complementary antimicrobial strategy, offering opportunities for improved infection control and innovative sterilization technologies to mitigate hospital-acquired infections.

## Introduction

Electromagnetic fields (EMFs) significantly influence biological systems, including microorganisms critical to human health and environmental processes. With the proliferation of radiofrequency and microwave radiation from technological sources, the exposure of microorganisms to electromagnetic radiation (EMR) has increased^1,2^. Among the various types of electromagnetic waves, millimeter waves (MMWs) for electromagnetic fields of extremely high frequencies, typically ranging from 30 GHz to 300 GHz, have received considerable attention because of their extensive use in communications, medical devices, and industrial applications^3,4^. As these technologies expand, there is a growing need to investigate the biological effects of High-frequency electromagnetic waves (HFEMWs), especially their impact on microorganisms, which are essential to human health and environmental processes^5,6^.

Studies have shown that MMWs can influence cellular functions, particularly bacteria, thereby impacting bacterial growth and antimicrobial activity. MMWs can induce various physical and biological effects, such as thermal heating, molecular excitation, and alterations in cell membrane permeability. The biological effects of these frequencies extend beyond thermal impacts, involving non-thermal mechanisms such as molecular excitation and changes in cell membrane permeability^7^. These properties make MMWs intriguing for microbial control, especially in light of the rising challenges of antibiotic resistance, suggesting that MMWs could increase the effectiveness of existing antibiotics or serve as alternative antimicrobial strategies^9^.

Recent studies have demonstrated that exposure to EHF radiation can modulate bacterial behavior, influencing growth rates, biofilm formation, and antibiotic sensitivity^10^. For instance, MMWs have been shown to enhance the efficacy of antibiotics by disrupting bacterial resistance mechanisms, offering a potential solution to the growing challenge of multidrug-resistant (MDR) pathogens^11^.

*Escherichia coli* and *Staphylococcus aureus* are two extensively studied bacterial species, highly relevant to public health due to their pathogenicity and increasing resistance to antibiotics. *E. coli*, a gram-negative bacterium commonly found in the human intestine, can cause severe infections if it is translocated to other body sites. *S. aureus*, a gram-positive bacterium, is known for its role in skin infections, pneumonia, and food poisoning and its increasing resistance to antibiotics^12^.

Resistance to antibiotics is evolving across a wide range of microorganisms, including both gram-positive and gram-negative bacteria, fungi, and even some viruses. Many bacterial species, including *Escherichia coli*, *Klebsiella pneumoniae*, *Pseudomonas aeruginosa*, and *Enterococcus faecium*, are developing resistance to multiple antibiotics. Infections caused by multidrug-resistant (MDR) are rising in both community and hospital settings, complicating treatment and leading to higher morbidity and mortality rates^13,15^.

*S. aureus* has developed resistance to many antibiotic classes, including macrolides, fluoroquinolones, and even vancomycin, which was once considered a last-resort antibiotic. The bacterium’s ability to form biofilms, particularly on medical devices, further enhances its resistance by protecting the bacterial community from antibiotic exposure and the immune system. The widespread resistance observed across bacterial species has become one of the most pressing issues in infectious disease treatment, requiring urgent development of new antibiotics and alternative treatment methods^14^.

Antibiotics, which are traditionally effective in treating serious infections, are becoming less effective because of bacterial mechanisms that evade their action, resulting in treatment failure and persistent infections^16^. This trend highlights the urgent need for new antimicrobial approaches and the careful use of existing antibiotics to prevent further resistance^8^. Investigating how high-frequency electromagnetic radiation interacts with bacteria such as *E. coli* and *S. aureus* is crucial, especially as these bacteria are frequently exposed to various forms of electromagnetic radiation (EMR) in medical environments. According to previous research, exposure to HFEMWs can dramatically change bacterial growth rates and inhibitory zone widths. Studies suggest that HFEMW radiation could mitigate these challenges by altering bacterial metabolism and membrane integrity, thereby increasing antibiotic sensitivity^17^. These results have important implications for managing severe infectious diseases and suggest that HFEMWs could enhance the efficacy of current antibiotics or offer alternative antimicrobial solutions^9,18,19^.

Furthermore, HFEMWs can be used in the sterilization of medical equipment, surfaces, or environments within hospitals. Maintaining a sterile environment is crucial for preventing infections, where patients may be immunocompromised due to treatments such as chemotherapy or radiation therapy. Understanding how EHF radiation affects bacterial survival could lead to new sterilization technologies, reducing the risk of hospital-acquired infections and improving patient outcomes. Despite these promising findings, the mechanisms underlying HFEMW-bacterial interactions remain poorly understood, and systematic investigations into frequency-dependent effects are limited^20^.

This study aimed to evaluate the effects of HFEMW on the growth rates of *E. coli* and *S. aureus*, as well as their responses to various antibiotics, including ceftazidime (3rd generation cephalosporin), ceftaroline (5th generation cephalosporin), gentamycin, doxycycline, and ciprofloxacin. Antibiotic sensitivity was assessed via the standard disk diffusion method according to the Clinical and Laboratory Standards Institute (CLSI, 2014) guidelines^21^, whereas the bacterial concentration was measured via absorbance at 600 nm and correlated with the number of colony-forming units on solid media^22^. The study revealed that exposure to HFEMW significantly affected the inhibition zone diameter and bacterial growth rate, suggesting potential implications for managing severe infections.

## Materials and methods

### Bacterial isolates

In this study, *Escherichia coli* (K-12) and *Staphylococcus aureus* (ATCC 29213) were obtained from the American Type Culture Collection (ATCC). The bacterial cultures were initially cultivated on agar plates (Oxoid, Hampshire, UK) and incubated aerobically at 37 °C for 24 h^23^. Pure colonies were then suspended in Mueller Hinton broth (Oxoid, Hampshire, UK) to achieve an optical density of 0.1 at 600 nm, ensuring standardized experimental conditions. For short-term preservation, a single bacterial colony was isolated, streaked onto a nutrient agar slant, incubated at 37 °C for 24 h, and subsequently stored at 4 °C^24^.

### Exposure to an electromagnetic field

**Fig. 1:**
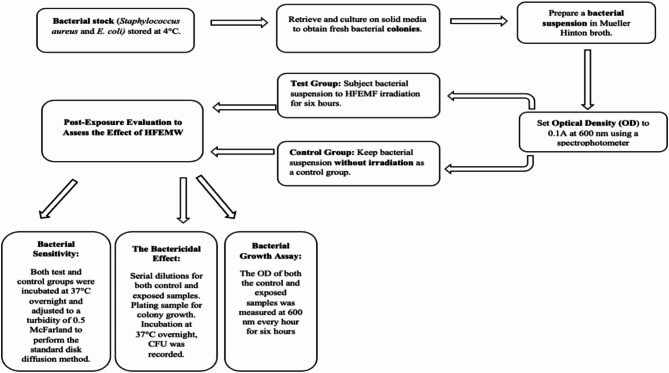
Block diagram for the experimental procedure investigating the effect of HFEMF on *Escherichia coli* and *Staphylococcus aureus.*.

As shown in Fig. 1, the bacterial cultures were irradiated via an electromagnetic radiation generator based on the backward-wave oscillator G4-141, which features a conical antenna that emits temporally coherent, nonradiative light, the distance from the antenna to the bacterial suspension was about 20 cm^25^. The polarized electromagnetic waves utilized had a wavelength range of 5.6–6.7 mm and a frequency range of 45–53 GHz^25^. This generator was developed by the Electrical Engineering Department at the Faculty of Engineering, Mansoura University. The bacterial cultures were exposed to this electromagnetic radiation at various frequencies for six hours. The system was housed within an incubator, maintaining a constant temperature of 37.0 ± 0.5 °C, verified by a thermometric sensor (Fluke 51-II, Fluke, WAQ3). This temperature was chosen to simulate physiological conditions for the bacteria.

### Antimicrobial susceptibility testing

Bacterial sensitivity was assessed via the standard disk diffusion method following Clinical and Laboratory Standards Institute (CLSI, 2014) guidelines^21^. The antibiotics selected for testing were chosen on the basis of their distinct mechanisms of action and included various classes, such as 3rd- and 5th-generation cephalosporins, aminoglycosides, fluoroquinolones, tetracyclines, and quinolones. Antibiotic disks from Oxoid (Hampshire, UK), including Ceftazidime (CAZ, 30 µg), Ceftaroline (CPT, 30 µg), Gentamycin (CN, 10 µg), doxycycline (DO, 30 µg), and ciprofloxacin (CIP, 5 µg), were used.

Test tubes containing bacterial cultures that were exposed to six different high frequencies (900 MHz, 1800 MHz, 51.8 GHz, 53 GHz, 70.6 GHz, and 73 GHz) for six hours, were incubated at 37 °C overnight, and adjusted to a turbidity of 0.5 McFarland standards. Another set of tubes was incubated at 37 °C overnight without exposure to the HFEMWs. The surfaces of the dried Mueller‒Hinton agar plates (Oxoid, Hampshire, UK) were inoculated in three directions via a cotton swab, and the antibiotic disks were placed with alcohol-filled fine-pointed forceps. The plates were incubated in an inverted position at 37 °C for 16–18 h. Inhibition zones were measured and categorized as resistant (R), intermediate (I), or sensitive (S) on the basis of CLSI guidelines^21^.

### Bacterial growth assay

The bacterial cultures were inoculated into fresh nutrient broth (Oxoid, Hampshire, UK) and incubated for 18 h to achieve late logarithmic phase growth. The bacteria were then transferred to 100 mL of fresh nutrient broth, and the optical density (OD) was adjusted to 0.1 at 600 nm to reach a concentration of 1 × 10^6^ colony-forming units (CFUs) per mL. One set of flasks was irradiated at the different tested frequencies, whereas an unexposed set served as a control^22^.

The OD of both the control and exposed samples was measured at 600 nm every hour for six hours, starting two hours after the beginning of irradiation, which represents the beginning of the log phase with measurements taken from three separate aliquots to ensure precision. In the end, a growth curve was drawn^26^.

### Statistical analysis

All experiments were replicated at least three times, and the statistical significance of each difference observed among the mean values was determined via standard error analysis. One-way ANOVA was used for statistical analysis via GraphPad Prism software (version 7.0; GraphPad Software Inc., La Jolla, CA, USA); *P <* 0.05 was considered to indicate statistical significance. All the data are expressed as the means ± SEMs.

## Results

### Effect of HFEMW on the sensitivity of bacteria to antibiotics

**Table 1:** Inhibition zone diameters (mm) and corresponding bacterial sensitivity of *E. Coli* cultures.

Antibiotics		Electromagnetic Exposure					
	Control	900 MHz	1800 MHz	51.8 GHz	53 GHz	70.6 GHz	73 GHz
CAZ	17/R	17/R	16/R	27/S	29/S	17/R	17/R
CPT	18/R	17/R	18/R	29/S	31/S	22/I	22/I
CN	10/R	11/R	12/R	19/S	20/S	10/R	11/R
DO	9/R	9/R	10/R	20/S	24/S	9/R	12/I
CIP	10/R	12/R	10/R	23/S	25/S	10/R	12/R

*R* Resistant, *I* Intermediate, *S* Sensitive, *CAZ* Ceftazidime, *CPT* Ceftaroline, *CN* Gentamycin, *DO* doxycycline, *CIP* ciprofloxacin.

**Table 2:** Inhibition zone diameter (mm) and corresponding bacterial sensitivity of *Staph. Aureus* cultures.

Antibiotics		Electromagnetic exposure					
	Control	900 MHz	1800 MHz	51.8 GHz	53 GHz	70.6 GHz	73 GHz
CAZ	15/R	15/R	16/R	24/S	26/S	17/R	16/R
CPT	22/I	23/I	23/I	30/S	32/S	29/S	29/S
CN	10/R	10/R	11/R	17/S	19/S	11/R	11/R
DO	14/I	14/I	15/I	19/S	22/S	12/I	16/S
CIP	13/R	14/R	15/R	25/S	25/S	15/R	14/R

*R* Resistant, *I* Intermediate, *S* Sensitive, *CAZ* Ceftazidime, *CPT* Ceftaroline, *CN* Gentamycin, *DO* doxycycline, *CIP* ciprofloxacin.

In this study, we investigated how high-frequency electromagnetic radiation affects the susceptibility of *E. coli* and *Staphylococcus* aureus to various antibiotics; the bacteria are more sensitive if their inhibition zone is greater. As shown in Table 1, *E. coli* was resistant to all the tested antibiotics, including 5th -generation ceftaroline. After exposure to different high electromagnetic frequencies ranging from 900 MHz to 73 GHz, the diameter of the inhibition zones increased after exposure to 51.8 GHz and 53 GHz for all the tested antibiotics, and the bacterial response shifted from resistant to sensitive. The largest inhibition zone was detected after exposure to 53 GHz, which was equal to 32 mm. Neither 900 MHz nor 1800 MHz showed any significant change. On the other hand, *E. coli* showed intermediate resistance to both ceftaroline and doxycycline after exposure to 73 GHz.

Similarly, for *Staphylococcus aureus* cultures, the diameters of the inhibition zones did not significantly change after exposure to 900 MHz and 1800 MHz. Moreover, bacteria became sensitive to all the tested antibiotics after exposure to both 51.8 GHz and 53 GHz. For the antibiotic ceftaroline, the inhibition zone increased from 22 mm to 29 mm after exposure to 70.6 GHz and 73 GHz, and *Staphylococcus aureus* became sensitive to doxycycline at 73 GHz, where the inhibition zone increased from 14 mm to 16 mm (Table 2).

These results indicated that, compared with the nonirradiated control, direct irradiation of bacteria with frequencies of 51.8, 53, 70.6, and 73 GHz decreased bacterial resistance to those different classes of antibiotics. The effects were more obvious at 51.8 GHz and 53 GHz than at 70.6 GHz and 73 GHz. Furthermore, our results demonstrated that there was no discernible impact of radiation exposure at 900 MHz or 1800 MHz on the size of the inhibition zone produced.

### The bactericidal effect of the HFEMW

**Fig. 2:**
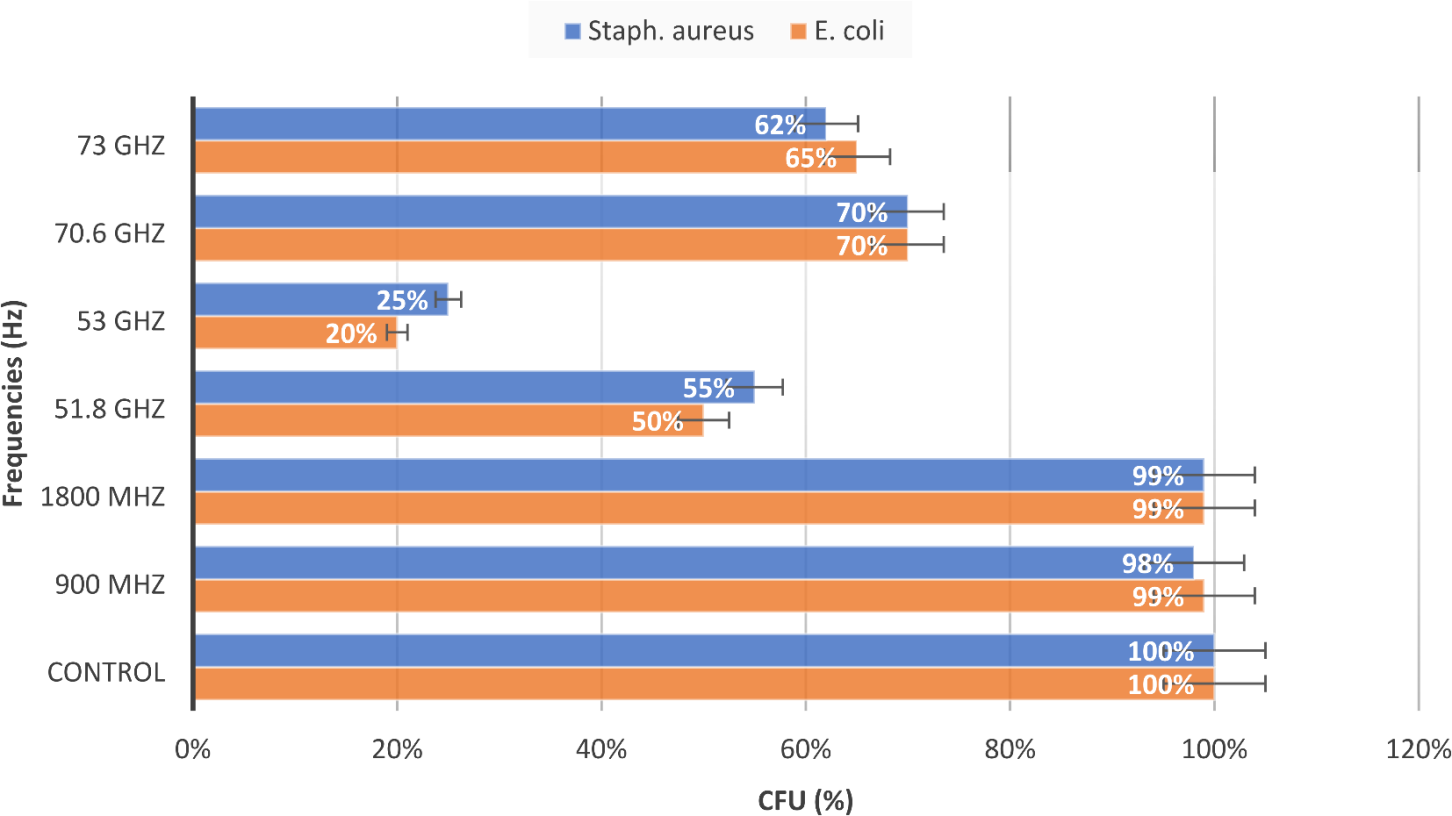
Changes in the percentages of *E. coli* and *Staphylococcus aureus* CFUs after irradiation with the HFEMW. Data represent the means ± SEMs from 3 different experiments.

*Escherichia coli* and *Staphylococcus aureus* cultures were irradiated at a wide range of frequencies from 900 MHz to 73 GHz, and the irradiation lasted for six hours. The colony-forming unit (CFU) values of the irradiated and control samples were measured under the chosen physical conditions. Large CFU numbers indicated a lower relative change in bacterial growth. Similarly, a lower number of CFUs indicated that the HFEMW had the greatest impact on bacterial growth, accounting for a greater relative change (%) value. As shown in Fig. 2, our results revealed the maximum percentage change for both *E. coli* and *Staphy. aureus* (20% and 25%) was observed at 53 GHz, followed by 50% and 55% at 51.8 GHz. A lower percentage change was observed after irradiation at 70.6 GHz and 73 GHz, ranging from 62 to 70%. The CFU counts were almost the same as those of the control after exposure to 900 MHz and 1800 MHz.

### Effect of the HFEMW on bacterial growth

Bacterial growth was estimated by measuring the optical density of the exposed sample via a spectrophotometer, the absorbance was measured at 600 nm every hour, and a calibration curve relating the absorbance to time was drawn.

The data presented in Tables 3 and 4 revealed that high-frequency electromagnetic radiation had a strong effect on bacterial growth, as represented by the optical density. Concerning the *E. coli* samples, our results revealed that the lowest absorbance was recorded at 53 GHz, where the OD decreased from 1.354 to 0.869, followed by exposure to 51.8 GHz. Furthermore, our results demonstrated that exposure to 70.6 GHz and 73 GHz had a weaker inhibitory effect on bacterial growth, with ODs of 1.172 and 1.169, respectively. On the other hand, there was almost no change in the OD values recorded after irradiation at 900 MHz and 1800 MHz.

Similarly, for *Staphylococcus aureus*, exposure to 900 MHz and 1800 MHz had no statistically significant effect. On the other hand, after radiation at 51.8 GHz and 53 GHz for six hours, the OD decreased from 1.614 to 1.134 and 1.090, respectively. The lowest P value (0.015) was calculated after exposure to 51.8 GHz and 53 GHz, followed by (0.026) at 70.6 GHz and 73 GHz, indicating a significant electromagnetic inhibitory effect on both *E. coli* and *Staphylococcus aureus* growth.

**Table 3:** Average optical density (OD) values for *E. Coli* culture exposed to the HFEMW.

Time (h)	Average (OD) values at different electromagnetic exposure						
	Control	900 MHz	1800 MHz	51.8 GHz^*^	53 GHz^*^	70.6 GHz^*^	73 GHz^*^
2	0.126	0.125	0.125	0.034	0.044	0.071	0.075
3	0.415	0.415	0.414	0.095	0.089	0.175	0.167
4	0.954	0.953	0.953	0.232	0.233	0.485	0.48
5	1.195	1.195	1.195	0.654	0.656	0.986	0.99
6	1.354	1.354	1.354	0.871	0.869	1.172	1.169

Statistical significance was determined using a one-way ANOVA. *p* < 0.05 is indicated by one asterisk (*).

**Table 4:** Average optical density (OD) values for *staph. Aureus* culture exposed to the HFEMW.

Time (h)	Average (OD) values at different electromagnetic exposure						
	Control	900 MHz	1800 MHz	51.8 GHz^*^	53 GHz^*^	70.6 GHz^*^	73 GHz^*^
2	0.201	0.202	0.203	0.109	0.111	0.145	0.140
3	0.462	0.462	0.462	0.143	0.140	0.223	0.209
4	0.987	0.986	0.986	0.264	0.262	0.519	0.502
5	1.449	1.450	1.448	0.909	0.889	1.243	1.255
6	1.614	1.614	1.615	1.134	1.090	1.435	1.420

Statistical significance was determined using a one-way ANOVA. *p* < 0.05 is indicated by one asterisk (*).

**Fig. 3:**
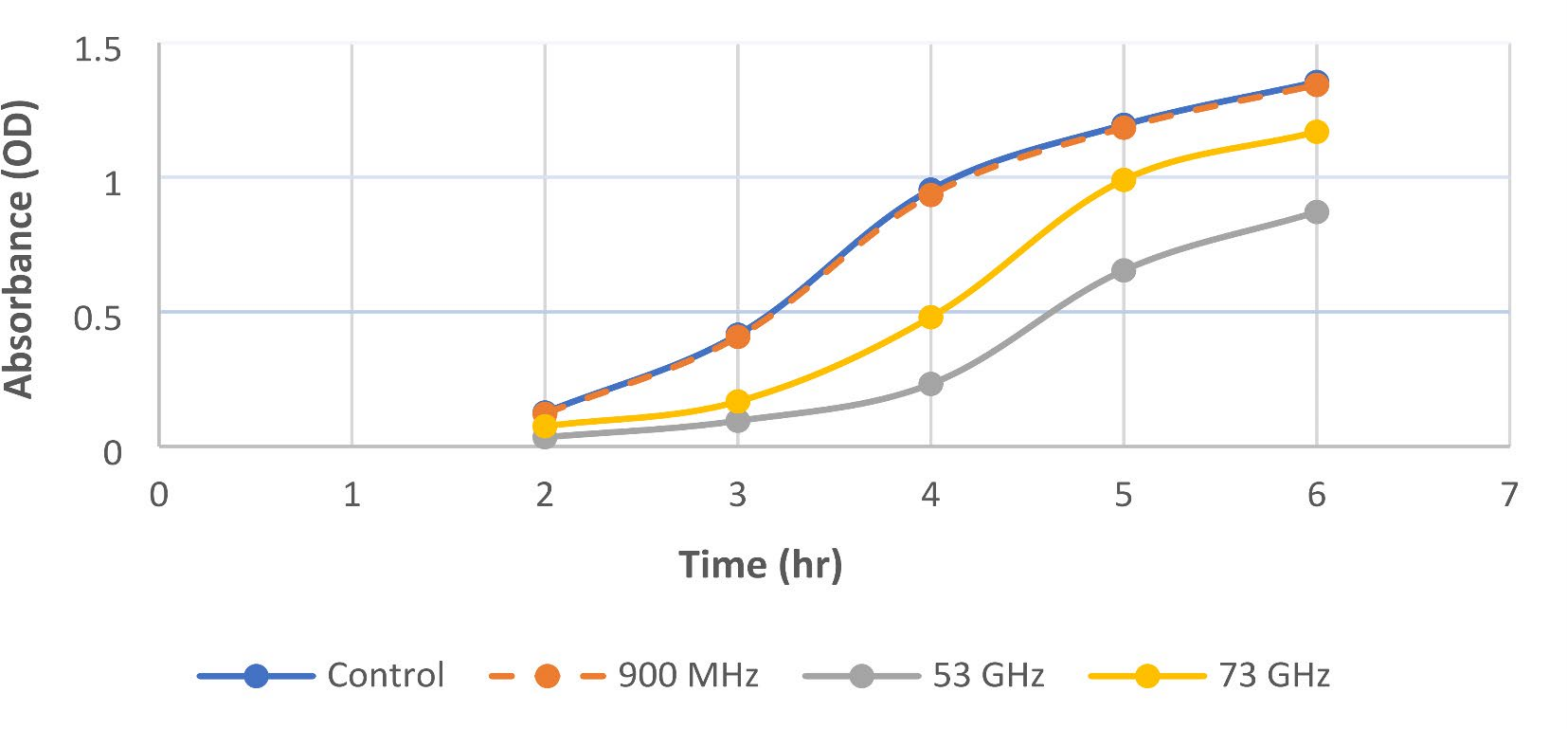
Effects of a representative HFEMW (900 MHz, 53 GHz, 73 GHz) on the optical density of *E. coli* samples compared with that of the control. Data represent the means ± SEMs from 3 different experiments.

Figure 3 showed the effects of exposure to electromagnetic fields at 900 MHz, 53 GHz, and 73 GHz on *E. coli* growth and that the optical density was increasing over time, which represented the log phase. However, exposing bacteria to high-frequency electromagnetic fields had an inhibitory effect on bacterial growth. Notably, exposing the bacteria to 900 MHz did not substantially affect the growth rate. While increasing the frequency to 73 GHz had a greater effect, the maximum decrease in the OD values was observed after exposure to 53 GHz.

## Discussion

Millimeter waves (MMWs) for electromagnetic fields of extremely high frequencies in the 30–300 GHz range are increasingly common because of their use in technologies such as satellite communication and radar^27^. Despite their nonthermal nature, these MMWs can significantly affect living organisms, increasing their ability to detect these organisms. Even low MMWs exposure levels can impact cells and bacteria, with applications in therapies, disinfection, and food safety^19^. In the food industry, MMWs help inactivate pathogens at low temperatures, preserving nutritional quality. The biological effects of these frequencies remain a critical concern^20^.

The pronounced effects of MMWs at the specified frequencies on *Escherichia coli*, a well-characterized bacterium and widely used model organism, have been thoroughly demonstrated. These effects become evident within tens of minutes following EMF exposure, with the optimal exposure duration being approximately one hour. In contrast, other MMW frequencies, such as 41–43 GHz, 61 GHz, and 99 GHz, do not significantly affect *E. coli*^10,11^. On the other hand, the effects of EMF can be amplified with prolonged exposure. This phenomenon has been observed at the 99 GHz frequency, where the impact of the EMF accumulates within the cell and becomes apparent at extended exposure durations^10^.

In this study, the results revealed that high-frequency millimeter waves of electromagnetic radiation (51.8 GHz to 73 GHz) had an overall inhibitory effect on bacterial growth. These results are in agreement with previous studies that demonstrated that the effects of EMF on *E. coli* suspensions are strongly evident at specific frequencies (50.3, 51.8, 64.5, 65.5, 95, and 105 GHz), which correspond to the resonant frequencies of H₂O molecules, suggesting that these effects are mediated by water^20,25,30^.

Our results revealed the maximum percentage change in CFU number for both *E. coli* and *Staph. aureus* was observed at 53 GHz (20% and 25%), followed by 50% and 55% at 51.8 GHz. On the other hand, a lower percentage change was observed after irradiation at 70.6 GHz and 73 GHz. These findings are in agreement with those of Torgomyan et al. (2011), who demonstrated that the growth of *E. coli* after the irradiation of bacterial suspensions in bidistilled water was more pronounced at 51.8 GHz and 53 GHz than at 70.6 GHz and 73 GHz. Furthermore, the effects of 70.6 GHz and 73 GHz on *E. coli* growth were found to be insignificant when the irradiated medium was used^8^.

Another study by Tadevosyan et al. (2007) revealed significant EMF-mediated effects on bacterial growth at 51.8 GHz and 53 GHz. Moreover, cell growth was similarly suppressed at 70.6 GHz and 73 GHz after direct irradiation of *E. coli* in either liquid or solid media. Interestingly, at 51.8 GHz and 53 GHz, the reduction in growth after *E. coli* suspension irradiation was less pronounced than that after direct bacterial irradiation on solid media, particularly at 53 GHz. The EMF interaction with H₂O may result in energy loss, with the amount of energy transferred depending on the specific frequency^8,10^.

The differential bactericidal effects of the MMW at frequencies of 51.8, 53, 70.6, and 73 GHz may be attributed to their primary resonant interactions with bacteria. This interaction can induce bacterial vibrations and alter metabolic activity, particularly those associated with bacterial membranes^31^. Compared with nonirradiated controls, direct irradiation of bacteria on solid growth medium at these frequencies resulted in a reduction in colony numbers, with more pronounced effects observed at 51.8 GHz and 53 GHz. Notably, MMW not only reduces the number of colonies but also affects their size^8^.

Antibiotics are known to cause modifications and disruptions of the bacterial cytoplasmic membrane, which manifest as changes in membrane properties, morphology, and biochemical processes^32,33^. *Escherichia coli* and *Staphylococcus aureus* have developed resistance to a variety of antibiotics. This resistance poses a significant challenge in clinical settings, where these antibiotics have traditionally been used to treat severe infections^34^. Ceftazidime, a third-generation cephalosporin, in addition to Ceftaroline, a fifth-generation cephalosporin, has been increasingly met with resistance due to the production of β-lactamases by these bacteria, which can degrade these antibiotics. Gentamycin, an aminoglycoside, is often compromised by enzymatic modification and efflux mechanisms in resistant strains. Doxycycline, a type of tetracycline, confers resistance through efflux pumps and ribosomal protection proteins. Ciprofloxacin, a fluoroquinolone, is less effective against resistant strains that carry mutations in DNA gyrase and topoisomerase IV or that utilize efflux mechanisms. This increasing resistance necessitates the exploration of alternative therapies and a deeper understanding of the mechanisms underlying bacterial resistance to ensure effective treatment strategies^35–37^.

Fortunately, recent studies have shown that 51.8 GHz and 53 GHz MMWs enhance antimicrobial activity against *Staphylococcus epidermidis*^38^. However, the modulation of antibiotic activity in different bacteria by low-intensity, extremely high-frequency EMF is a novel phenomenon that warrants further investigation and could have broad applications. In the present study, the diameters of the inhibition zones of the tested antibiotics increased after exposure to 51.8 GHz and 53 GHz, and the bacterial response shifted from resistant to sensitive, indicating that MMW exposure can modify antibiotic sensitivity or resistance in a frequency-dependent manner. Enhanced effects are particularly notable at 51.8 GHz and more so at 53 GHz. This conclusion was in agreement with a previous study stating that MMW at 41–43 GHz, 53 GHz, 70.6 GHz, and 73 GHz can exhibit resonant-like bactericidal effects on *E. coli*, likely through direct interactions with the bacteria^39^.

Additionally, other tested antibiotics, including tetracycline, chloramphenicol, kanamycin, and ceftriaxone, at their minimal inhibitory concentrations, were found to influence *E. coli* growth and survival, and these effects can be potentiated by extremely high-frequency EMF^8,10,25^. Some explanations for the alteration in bacterial sensitivity to antibiotics were mentioned by Fojt et al. (2010), who hypothesized that membrane permeabilization; disruption of the proton‒motive force generated by the F0F1-ATPase under fermentation conditions; disturbances in H⁺, K⁺, and Na⁺ transport; alterations in transport systems; and a reduction in ATP levels as well as changes in the membrane proteome can increase bacterial sensitivity to antibiotics^40^. Additionally, modifications in DNA gyrase and other changes in gene expression or protein synthesis are critical factors influencing antibiotic sensitivity. These findings are consistent with the observed changes in *Staphylococcus aureus* sensitivity to various antibiotics when the bacteria are exposed to extremely high-frequency EMFs with nonthermal intensity^11^.

On the other hand, the results generally indicated that exposure to 900 MHz and 1800 MHz radiation did not significantly affect the size of the inhibition zones produced by the various antibiotics tested or the growth rate or the CFU values. Except for a significant decrease after 12 h of exposure to 900 MHz, no significant effects on the growth of *S. aureus* or *S. epidermidis* were observed. These studies concluded that the inhibitory effects on bacterial growth increase with increasing duration of exposure^40,41^.

## Conclusion

In this study, pathogenic bacteria, including *Escherichia coli* and *Staphylococcus aureus*, were exposed to high-frequency electromagnetic radiation, and the subsequent effects on the bacterial growth rate and antibiotic sensitivity were evaluated. The inhibition zone diameter, bacterial growth, and number of colony-forming units (CFUs) were measured at an optical density of 600 nm. Our results revealed no significant differences compared to the control following exposure to 900 and 1800 MHz. However, exposure to 70.6 GHz and 73 GHz resulted in observable changes in these parameters. Notably, at 51.8 GHz and 53 GHz, clear electromagnetic interference (EMI) effects were detected, with the 53 GHz frequency producing more pronounced results. These findings highlight the varying effects of different EMF frequencies on bacterial viability and underscore the complexity of interactions between environmental conditions and EMF parameters in influencing bacterial growth outcomes.

## Data Availability

Data will be available upon request through Dr/ May El-Antrawy email: mayantrawy92@gmail.com.

## References

[1] Alipov, E., Shcheglov, V., Sarimov, R. & Belyaev, I. Y. Cell-density dependent effects of low-dose ionizing radiation on E. Coli cells. *Radiats. Biol. Radioecol.* **43** (2), 167–171 (2003). 12754801

[2] Salmen, S. H., Alharbi, S. A., Faden, A. A. & Wainwright, M. Evaluation of effect of high frequency electromagnetic field on growth and antibiotic sensitivity of bacteria. *Saudi J. Biol. Sci.* **25** (1), 105–110 (2018). 29379365 10.1016/j.sjbs.2017.07.006 PMC5775109

[3] Uwaechia, A. N. & Mahyuddin, N. M. A comprehensive survey on millimeter wave communications for fifth-generation wireless networks: feasibility and challenges. *IEEE Access* **8**, 62367–62414 (2020).

[4] Wang, X. et al. Millimeter wave communication: a comprehensive survey. *IEEE Commun. Surv. Tutor.* **20** (3), 1616–1653 (2018).

[5] Jing, R., Jiang, Z. & Tang, X. Advances in millimeter-wave treatment and its biological effects development. *Int. J. Mol. Sci.* **25** (16), 8638 (2024). 39201326 10.3390/ijms25168638 PMC11354414

[6] Mattsson, M. O., Zeni, O. & Simkó, M. Is there a biological basis for therapeutic applications of millimetre waves and THz waves?. *J. Infrared Millim. Terahertz Waves*. **39** (9), 863–878 (2018).

[7] Pakhomov, A. G. & Murphy, M. R. A comprehensive review of the research on biological effects of pulsed radiofrequency radiation in Russia and the former Soviet Union. *Advances in electromagnetic fields in living systems* 265–290 (2000).

[8] Torgomyan, H., Tadevosyan, H. & Trchounian, A. Extremely high frequency electromagnetic irradiation in combination with antibiotics enhances antibacterial effects on Escherichia coli. *Curr. Microbiol.* **62**, 962–967 (2011). 21079961 10.1007/s00284-010-9811-2

[9] Trushin, M. V. The possible role of electromagnetic fields in bacterial communication. *J. Microbiol. Immunol. Infect.* **36** (3), 153–160 (2003). 14582558

[10] Torgomyan, H. & Trchounian, A. Bactericidal effects of low-intensity extremely high frequency electromagnetic field: an overview with phenomenon, mechanisms, targets and consequences. *Crit. Rev. Microbiol.* **39** (1), 102–111 (2013). 22667685 10.3109/1040841X.2012.691461

[11] Bulgakova, V. et al. The effect of millimeter-band radiation of nonthermal intensity on sensitivity of Staphylococcus to various antibiotics. *Biofizika* **41** (6), 1289–1293 (1996). 9044624

[12] Kadry, A. A., El-Antrawy, M. A. & El-Ganiny, A. M. Management of clinical infections of Escherichia coli by new β-lactam/β-lactamase inhibitor combinations. *Iran. J. Microbiol.* **14** (4), 466 (2022). 36721515 10.18502/ijm.v14i4.10232 PMC9867644

[13] Yang, J. et al. Exploring multidrug-resistant Klebsiella pneumoniae antimicrobial resistance mechanisms through whole genome sequencing analysis. *BMC Microbiol.* **23** (1), 245 (2023). 37660028 10.1186/s12866-023-02974-y PMC10474722

[14] Peng, Q., Tang, X., Dong, W., Sun, N. & Yuan, W. A review of biofilm formation of Staphylococcus aureus and its regulation mechanism. *Antibiotics* **12** (1), 12 (2022). 36671212 10.3390/antibiotics12010012 PMC9854888

[15] Kadry, A. A., El-Antrawy, M. A. & El-Ganiny, A. M. Management of clinical infections of Escherichia coli by new β-lactam/β-lactamase inhibitor combinations. *Iran. J. Microbiol.* **14** (4), 466–474 (2022). 36721515 10.18502/ijm.v14i4.10232 PMC9867644

[16] Kadry, A. A., El-Antrawy, M. A. & El-Ganiny, A. M. Impact of short chain fatty acids (SCFAs) on antimicrobial activity of new β-lactam/β-lactamase inhibitor combinations and on virulence of Escherichia coli isolates. *J. Antibiot.* **76** (4), 225–235 (2023). 10.1038/s41429-023-00595-1 PMC10040337 36726014

[17] Shamis, Y., Croft, R., Taube, A., Crawford, R. J. & Ivanova, E. P. Review of the specific effects of microwave radiation on bacterial cells. *Appl. Microbiol. Biotechnol.* **96**, 319–325 (2012). 22875401 10.1007/s00253-012-4339-y

[18] Matsuhashi, M. et al. Production of sound waves by bacterial cells and the response of bacterial cells to sound. *J. Gen. Appl. Microbiol.* **44** (1), 49–55 (1998). 12501293 10.2323/jgam.44.49

[19] Balcavage, W. et al. A mechanism for action of extremely low frequency electromagnetic fields on biological systems. *Biochem. Biophys. Res. Commun.* **222** (2), 374–378 (1996). 8670212 10.1006/bbrc.1996.0751

[23] Betskii, O., Devyatkov, N. & Kislov, V. Low intensity millimeter waves in medicine and biology.* Crit. Rev. Biomed. Eng*.** 28**(1 & 2) (2000). 10.1615/critrevbiomedeng.v28.i12.420 10999395

[24] Clinical and Laboratory Standards Institute. Performance standards for antimicrobial disk susceptibility tests; approved standard—Twelfth Edition. CLSI document M02-A11 (2014).

[22] Zwietering, M. H., Jongenburger, I., Rombouts, F. M. & Van’t Riet, K. Modeling of the bacterial growth curve. *Appl. Environ. Microbiol.* **56** (6), 1875–1881 (1990). 16348228 10.1128/aem.56.6.1875-1881.1990 PMC184525

[23] Atlas, R. M. *Handbook of Microbiological Media* (CRC, 2004).

[24] Washington, C., Stephen, A. & Janda, W. *Koneman’s Color Atlas and Textbook of Diagnostic Microbiology* (Lippincott Williams & Wilkins, 2006).

[25] Tadevosyan, H., Kalantaryan, V. & Trchounian, A. Extremely high frequency electromagnetic radiation enforces bacterial effects of inhibitors and antibiotics. *Cell. Biochem. Biophys.* **51**, 97–103 (2008). 18633580 10.1007/s12013-008-9020-9

[26] Paulton, R. J. The bacterial growth curve. *J. Biol. Educ.* **25** (2), 92–94 (1991).

[27] Xiao, M. et al. Millimeter wave communications for future mobile networks. *IEEE J. Sel. Areas Commun.* **35** (9), 1909–1935 (2017).

[28] Trchunian, A. et al. Membranotropic effects of electromagnetic radiation of extremely high frequency on Escherichia coli. *Biofizika* **46** (1), 69–76 (2001). 11236565

[29] Cabiscol Catalā, E., Tamarit Sumalla, J. & Ros Salvador, J. Oxidative stress in bacteria and protein damage by reactive oxygen species (2000). 10963327

[30] Isakhanyan, V. & Trchunyan, A. Indirect and repeated extremely high frequency electromagnetic irradiation of bacteria Escherichia Coli. *Biophysics* **50** (4), 604–606 (2005). 16212062

[31] Shcheglov, V. S., Alipov, E. D. & Belyaev, I. Y. Cell-to-cell communication in response of E. coli cells at different phases of growth to low-intensity microwaves. *Biochim. Biophys. Acta (BBA)-Gen Subj.* **1572** (1), 101–106 (2002). 10.1016/s0304-4165(02)00283-0 12204338

[32] Lai, C. C., Chen, C. C., Huang, H. L., Chuang, Y. C. & Tang, H. J. The role of doxycycline in the therapy of multidrug-resistant E. coli–an in vitro study. *Sci. Rep.* **6** (1), 1–9 (2016). 27534373 10.1038/srep31964 PMC4989187

[33] Bao, W., Koro, M., Loh, G. W. & Ortiz, A. Urinary tract infections in long-term care: evaluation of uropathogens, antibiotic susceptibility, and empiric treatment. *Sr. Care Pharm.* **37** (6), 232–243 (2022). 35610764 10.4140/TCP.n.2022.232

[34] Ali, I., Rafaque, Z., Ahmed, S., Malik, S. & Dasti, J. I. Prevalence of multi-drug resistant uropathogenic Escherichia coli in Potohar region of Pakistan. *Asian Pac. J. Trop. Biomed.* **6** (1), 60–66 (2016).

[35] Abdul Rahim, N. et al. Synergistic killing of NDM-producing MDR Klebsiella pneumoniae by two ‘old’ antibiotics—polymyxin B and chloramphenicol. *J. Antimicrob. Chemother.* **70** (9), 2589–2597 (2015). 26023209 10.1093/jac/dkv135 PMC4635649

[36] Alfei, S. & Schito, A. M. β-Lactam antibiotics and β-lactamase enzymes inhibitors, part 2: our limited resources. *Pharmaceuticals* **15** (4), 476 (2022). 35455473 10.3390/ph15040476 PMC9031764

[37] Bassetti, M. & Garau, J. Current and future perspectives in the treatment of multidrug-resistant Gram-negative infections. *J. Antimicrob. Chemother.* **76**, IV23–37 (2021). 34849997 10.1093/jac/dkab352 PMC8632738

[38] Matewele, P. The effect of electromagnetic field on antimicrobial activity of lime oil. *J. Microbiol. Methods*. **83** (2), 275–276 (2010). 20863860 10.1016/j.mimet.2010.09.015

[39] Yu, G. et al. A study on biological effects of low-intensity millimeter waves. *IEEE Trans. Plasma Sci.* **30** (4), 1489–1496 (2002).

[40] Fojt, L., Strašák, L., Vetterl, V. & Šmarda, J. Comparison of the low-frequency magnetic field effects on bacteria Escherichia coli, Leclercia adecarboxylata and Staphylococcus aureus. *Bioelectrochemistry* **63** (1–2), 337–341 (2004). 15110299 10.1016/j.bioelechem.2003.11.010

[41] Gaafar, E. S. A., Hanafy, M. S., Tohamy, E. Y. & Ibrahim, M. H. Stimulation and control of E. coli by using an extremely low frequency magnetic field. *Roman J. Biophys.* **16** (4), 283–296 (2006).

